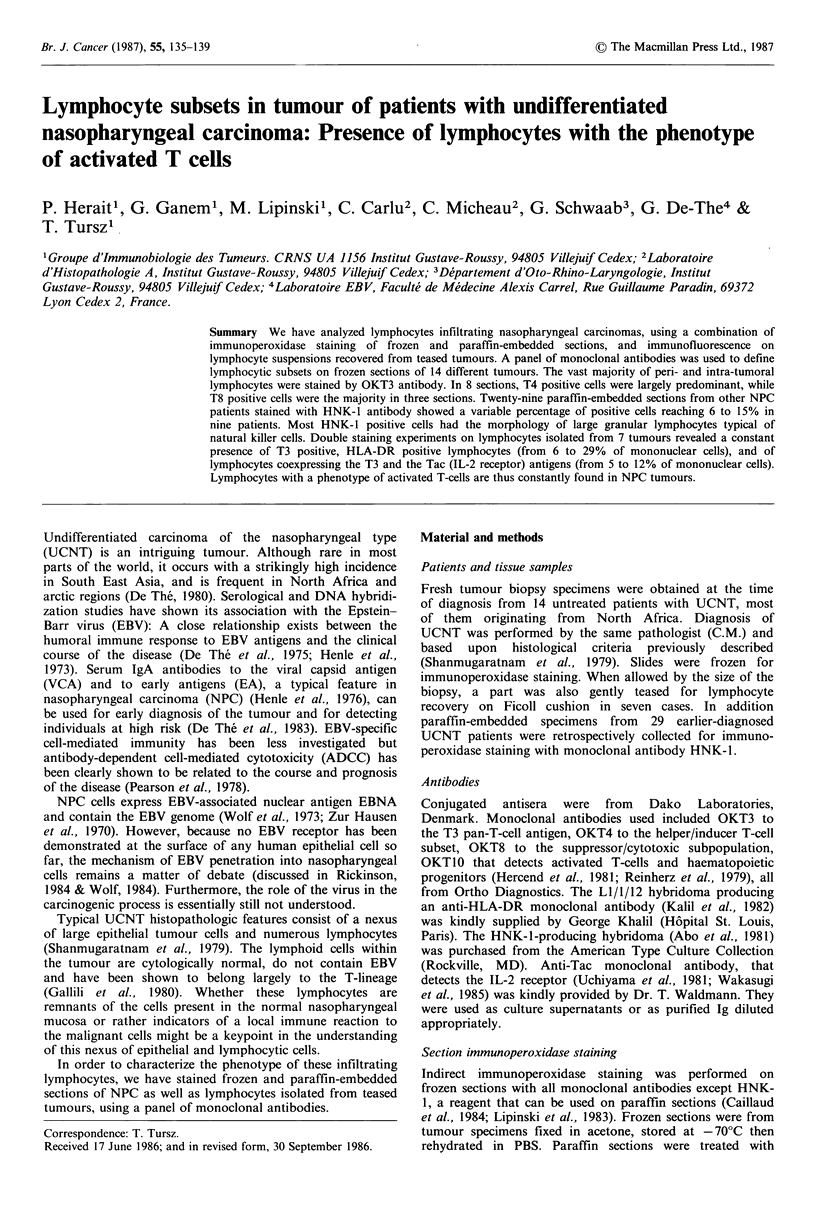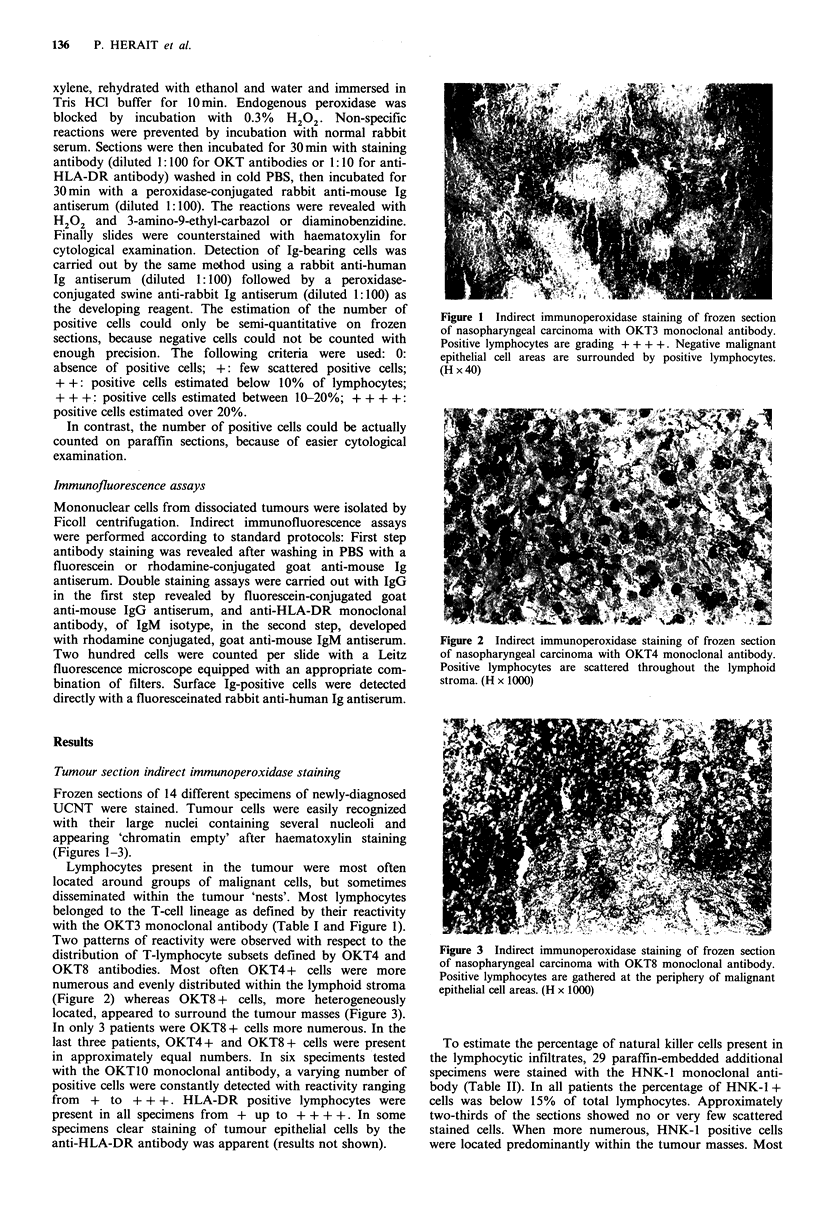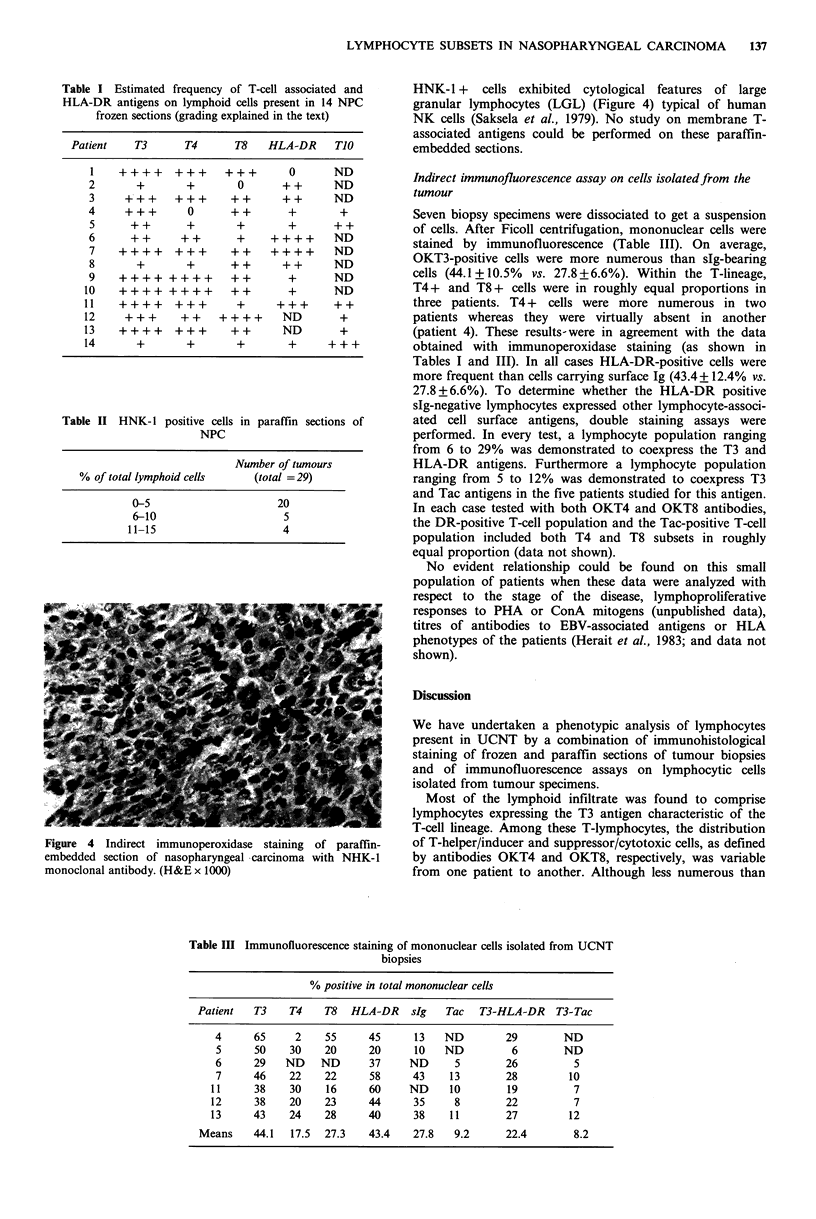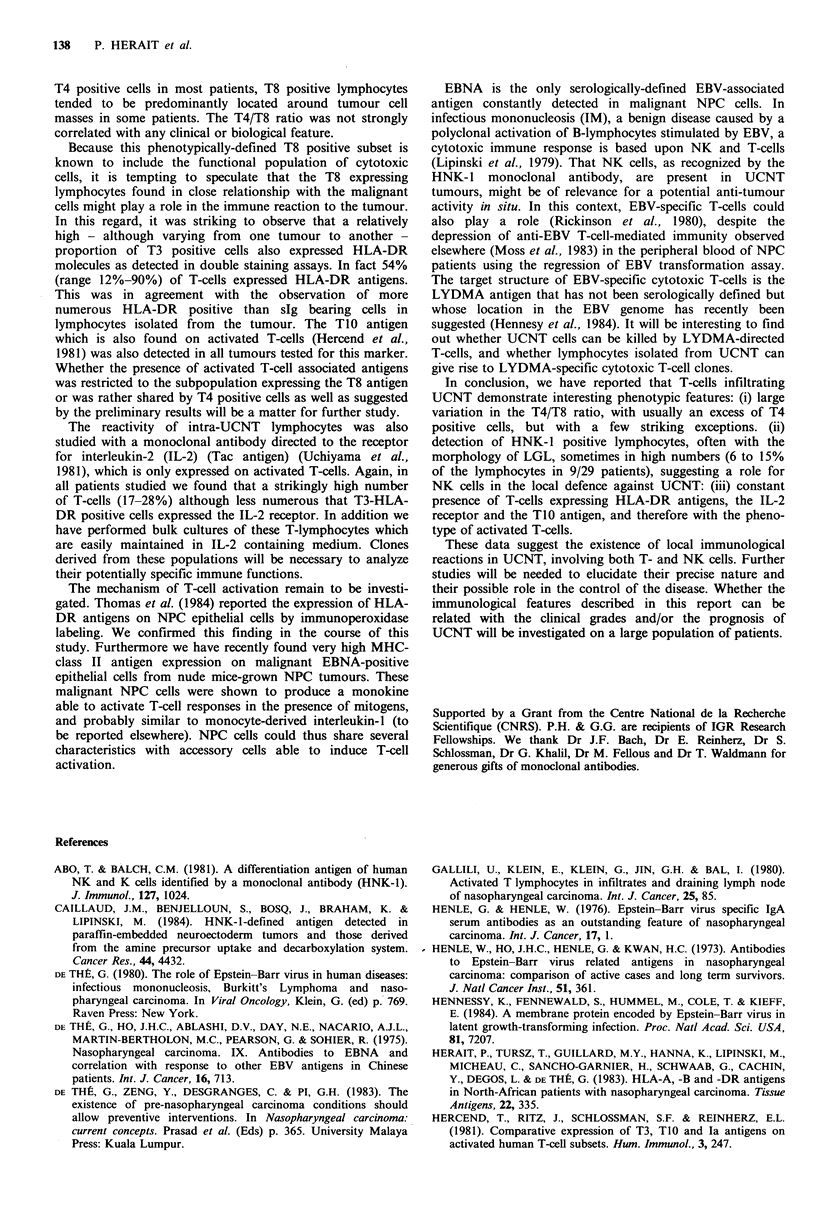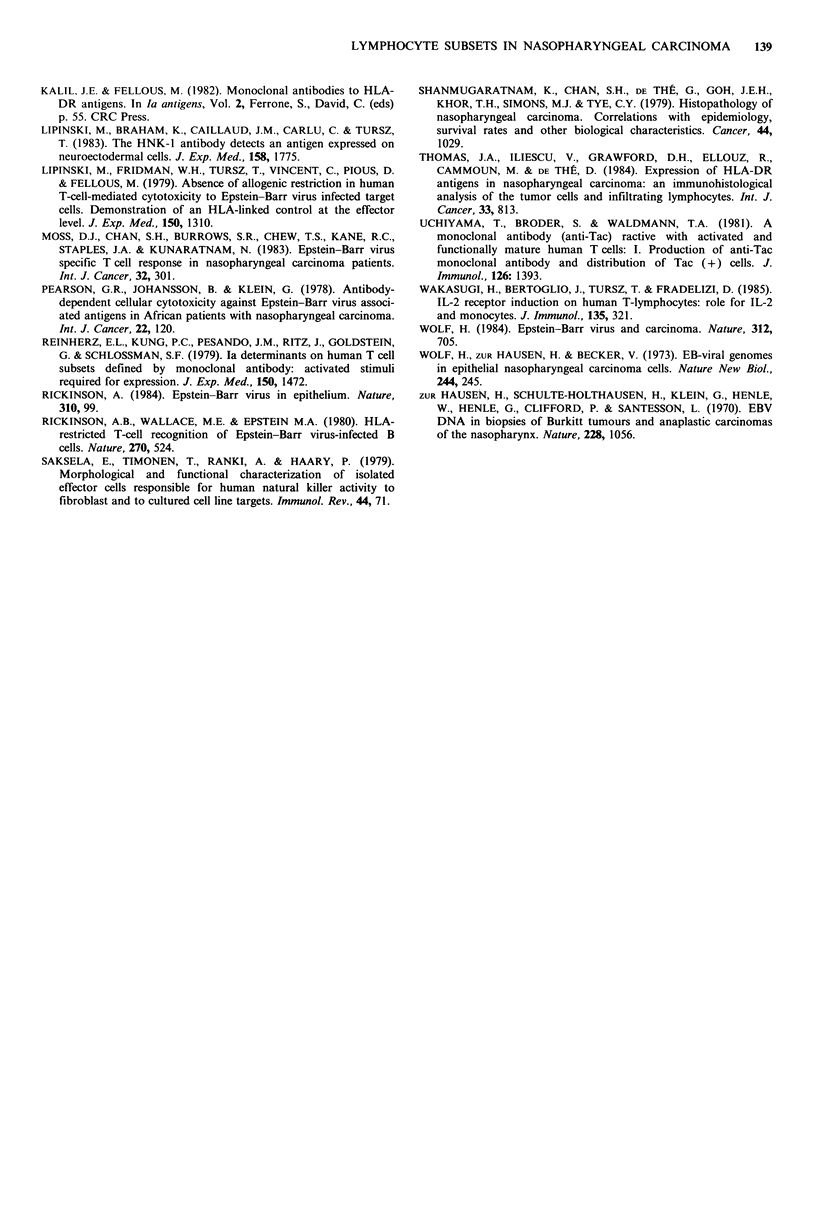# Lymphocyte subsets in tumour of patients with undifferentiated nasopharyngeal carcinoma: presence of lymphocytes with the phenotype of activated T cells.

**DOI:** 10.1038/bjc.1987.28

**Published:** 1987-02

**Authors:** P. Herait, G. Ganem, M. Lipinski, C. Carlu, C. Micheau, G. Schwaab, G. De-The, T. Tursz

## Abstract

**Images:**


*Br. J. Cancer* (1987), **55**, 135–139 © The Macmillan Press Ltd., 1987

### Lymphocyte subsets in tumour of patients with undifferentiated nasopharyngeal carcinoma: Presence of lymphocytes with the phenotype of activated T cells

P. Herait[1], G. Ganem[1], M. Lipinski[1], C. Carlu[2], C. Micheau[2], G. Schwaab[3], G. De-The[4] & T. Tursz[1]

[1]*Groupe d'Immunobiologie des Tumeurs. CRNS UA 1156 Institut Gustave-Roussy, 94805 Villejuif Cedex;* [2]*Laboratoire d'Histopathologie A, Institut Gustave-Roussy, 94805 Villejuif Cedex;* [3]*Département d'Oto-Rhino-Laryngologie, Institut Gustave-Roussy, 94805 Villejuif Cedex;* [4]*Laboratoire EBV, Faculté de Médecine Alexis Carrel, Rue Guillaume Paradin, 69372 Lyon Cedex 2, France.*

**Summary** We have analyzed lymphocytes infiltrating nasopharyngeal carcinomas, using a combination of immunoperoxidase staining of frozen and paraffin-embedded sections, and immunofluorescence on lymphocyte suspensions recovered from teased tumours. A panel of monoclonal antibodies was used to define lymphocytic subsets on frozen sections of 14 different tumours. The vast majority of peri- and intra-tumoral lymphocytes were stained by OKT3 antibody. In 8 sections, T4 positive cells were largely predominant, while T8 positive cells were the majority in three sections. Twenty-nine paraffin-embedded sections from other NPC patients stained with HNK-1 antibody showed a variable percentage of positive cells reaching 6 to 15% in nine patients. Most HNK-1 positive cells had the morphology of large granular lymphocytes typical of natural killer cells. Double staining experiments on lymphocytes isolated from 7 tumours revealed a constant presence of T3 positive, HLA-DR positive lymphocytes (from 6 to 29% of mononuclear cells), and of lymphocytes coexpressing the T3 and the Tac (IL-2 receptor) antigens (from 5 to 12% of mononuclear cells). Lymphocytes with a phenotype of activated T-cells are thus constantly found in NPC tumours.

Undifferentiated carcinoma of the nasopharyngeal type (UCNT) is an intriguing tumour. Although rare in most parts of the world, it occurs with a strikingly high incidence in South East Asia, and is frequent in North Africa and arctic regions (De Thé, 1980). Serological and DNA hybridization studies have shown its association with the Epstein–Barr virus (EBV): A close relationship exists between the humoral immune response to EBV antigens and the clinical course of the disease (De Thé *et al.*, 1975; Henle *et al.*, 1973). Serum IgA antibodies to the viral capsid antigen (VCA) and to early antigens (EA), a typical feature in nasopharyngeal carcinoma (NPC) (Henle *et al.*, 1976), can be used for early diagnosis of the tumour and for detecting individuals at high risk (De Thé *et al.*, 1983). EBV-specific cell-mediated immunity has been less investigated but antibody-dependent cell-mediated cytotoxicity (ADCC) has been clearly shown to be related to the course and prognosis of the disease (Pearson *et al.*, 1978).

NPC cells express EBV-associated nuclear antigen EBNA and contain the EBV genome (Wolf *et al.*, 1973; Zur Hausen *et al.*, 1970). However, because no EBV receptor has been demonstrated at the surface of any human epithelial cell so far, the mechanism of EBV penetration into nasopharyngeal cells remains a matter of debate (discussed in Rickinson, 1984 & Wolf, 1984). Furthermore, the role of the virus in the carcinogenic process is essentially still not understood.

Typical UCNT histopathologic features consist of a nexus of large epithelial tumour cells and numerous lymphocytes (Shanmugaratnam *et al.*, 1979). The lymphoid cells within the tumour are cytologically normal, do not contain EBV and have been shown to belong largely to the T-lineage (Gallili *et al.*, 1980). Whether these lymphocytes are remnants of the cells present in the normal nasopharyngeal mucosa or rather indicators of a local immune reaction to the malignant cells might be a keypoint in the understanding of this nexus of epithelial and lymphocytic cells.

In order to characterize the phenotype of these infiltrating lymphocytes, we have stained frozen and paraffin-embedded sections of NPC as well as lymphocytes isolated from teased tumours, using a panel of monoclonal antibodies.

Correspondence: T. Tursz.
Received 17 June 1986; and in revised form, 30 September 1986.

### Material and methods

### *Patients and tissue samples*

Fresh tumour biopsy specimens were obtained at the time of diagnosis from 14 untreated patients with UCNT, most of them originating from North Africa. Diagnosis of UCNT was performed by the same pathologist (C.M.) and based upon histological criteria previously described (Shanmugaratnam *et al.*, 1979). Slides were frozen for immunoperoxidase staining. When allowed by the size of the biopsy, a part was also gently teased for lymphocyte recovery on Ficoll cushion in seven cases. In addition paraffin-embedded specimens from 29 earlier-diagnosed UCNT patients were retrospectively collected for immunoperoxidase staining with monoclonal antibody HNK-1.

### *Antibodies*

Conjugated antisera were from Dako Laboratories, Denmark. Monoclonal antibodies used included OKT3 to the T3 pan-T-cell antigen, OKT4 to the helper/inducer T-cell subset, OKT8 to the suppressor/cytotoxic subpopulation, OKT10 that detects activated T-cells and haematopoietic progenitors (Hercend *et al.*, 1981; Reinherz *et al.*, 1979), all from Ortho Diagnostics. The L1/1/12 hybridoma producing an anti-HLA-DR monoclonal antibody (Kalil *et al.*, 1982) was kindly supplied by George Khalil (Hôpital St. Louis, Paris). The HNK-1-producing hybridoma (Abo *et al.*, 1981) was purchased from the American Type Culture Collection (Rockville, MD). Anti-Tac monoclonal antibody, that detects the IL-2 receptor (Uchiyama *et al.*, 1981; Wakasugi *et al.*, 1985) was kindly provided by Dr. T. Waldmann. They were used as culture supernatants or as purified Ig diluted appropriately.

### *Section immunoperoxidase staining*

Indirect immunoperoxidase staining was performed on frozen sections with all monoclonal antibodies except HNK-1, a reagent that can be used on paraffin sections (Caillaud *et al.*, 1984; Lipinski *et al.*, 1983). Frozen sections were from tumour specimens fixed in acetone, stored at −70°C then rehydrated in PBS. Paraffin sections were treated with xylene, rehydrated with ethanol and water and immersed in Tris HCl buffer for 10 min. Endogenous peroxidase was blocked by incubation with 0.3% $H_2O_2$. Non-specific reactions were prevented by incubation with normal rabbit serum. Sections were then incubated for 30 min with staining antibody (diluted 1:100 for OKT antibodies or 1:10 for anti-HLA-DR antibody) washed in cold PBS, then incubated for 30 min with a peroxidase-conjugated rabbit anti-mouse Ig antiserum (diluted 1:100). The reactions were revealed with $H_2O_2$ and 3-amino-9-ethyl-carbazol or diaminobenzidine. Finally slides were counterstained with haematoxylin for cytological examination. Detection of Ig-bearing cells was carried out by the same method using a rabbit anti-human Ig antiserum (diluted 1:100) followed by a peroxidase-conjugated swine anti-rabbit Ig antiserum (diluted 1:100) as the developing reagent. The estimation of the number of positive cells could only be semi-quantitative on frozen sections, because negative cells could not be counted with enough precision. The following criteria were used: 0: absence of positive cells; +: few scattered positive cells; + +: positive cells estimated below 10% of lymphocytes; + + +: positive cells estimated between 10–20%; + + + +: positive cells estimated over 20%.

In contrast, the number of positive cells could be actually counted on paraffin sections, because of easier cytological examination.

*Immunofluorescence assays*

Mononuclear cells from dissociated tumours were isolated by Ficoll centrifugation. Indirect immunofluorescence assays were performed according to standard protocols: First step antibody staining was revealed after washing in PBS with a fluorescein or rhodamine-conjugated goat anti-mouse Ig antiserum. Double staining assays were carried out with IgG in the first step revealed by fluorescein-conjugated goat anti-mouse IgG antiserum, and anti-HLA-DR monoclonal antibody, of IgM isotype, in the second step, developed with rhodamine conjugated, goat anti-mouse IgM antiserum. Two hundred cells were counted per slide with a Leitz fluorescence microscope equipped with an appropriate combination of filters. Surface Ig-positive cells were detected directly with a fluoresceinated rabbit anti-human Ig antiserum.

### Results

*Tumour section indirect immunoperoxidase staining*

Frozen sections of 14 different specimens of newly-diagnosed UCNT were stained. Tumour cells were easily recognized with their large nuclei containing several nucleoli and appearing 'chromatin empty' after haematoxylin staining (Figures 1–3).

Lymphocytes present in the tumour were most often located around groups of malignant cells, but sometimes disseminated within the tumour 'nests'. Most lymphocytes belonged to the T-cell lineage as defined by their reactivity with the OKT3 monoclonal antibody (Table I and Figure 1). Two patterns of reactivity were observed with respect to the distribution of T-lymphocyte subsets defined by OKT4 and OKT8 antibodies. Most often OKT4+ cells were more numerous and evenly distributed within the lymphoid stroma (Figure 2) whereas OKT8+ cells, more heterogeneously located, appeared to surround the tumour masses (Figure 3). In only 3 patients were OKT8+ cells more numerous. In the last three patients, OKT4+ and OKT8+ cells were present in approximately equal numbers. In six speciments tested with the OKT10 monoclonal antibody, a varying number of positive cells were constantly detected with reactivity ranging from + to + + +. HLA-DR positive lymphocytes were present in all specimens from + up to + + + +. In some specimens clear staining of tumour epithelial cells by the anti-HLA-DR antibody was apparent (results not shown).

**Figure 1** Indirect immunoperoxidase staining of frozen section of nasopharyngeal carcinoma with OKT3 monoclonal antibody. Positive lymphocytes are grading + + + +. Negative malignant epithelial cell areas are surrounded by positive lymphocytes. (H × 40)

**Figure 2** Indirect immunoperoxidase staining of frozen section of nasopharyngeal carcinoma with OKT4 monoclonal antibody. Positive lymphocytes are scattered throughout the lymphoid stroma. (H × 1000)

**Figure 3** Indirect immunoperoxidase staining of frozen section of nasopharyngeal carcinoma with OKT8 monoclonal antibody. Positive lymphocytes are gathered at the periphery of malignant epithelial cell areas. (H × 1000)

To estimate the percentage of natural killer cells present in the lymphocytic infiltrates, 29 paraffin-embedded additional specimens were stained with the HNK-1 monoclonal antibody (Table II). In all patients the percentage of HNK-1+ cells was below 15% of total lymphocytes. Approximately two-thirds of the sections showed no or very few scattered stained cells. When more numerous, HNK-1 positive cells were located predominantly within the tumour masses. Most

**Table I** Estimated frequency of T-cell associated and HLA-DR antigens on lymphoid cells present in 14 NPC frozen sections (grading explained in the text)

| *Patient* | *T3* | *T4* | *T8* | *HLA-DR* | *T10* |
|---|---|---|---|---|---|
| 1 | ++++ | +++ | +++ | 0 | ND |
| 2 | + | + | 0 | ++ | ND |
| 3 | +++ | +++ | ++ | ++ | ND |
| 4 | +++ | 0 | ++ | + | + |
| 5 | ++ | + | + | + | ++ |
| 6 | ++ | ++ | + | ++++ | ND |
| 7 | ++++ | +++ | ++ | ++++ | ND |
| 8 | + | + | ++ | ++ | ND |
| 9 | ++++ | ++++ | ++ | + | ND |
| 10 | ++++ | ++++ | ++ | + | ND |
| 11 | ++++ | +++ | + | +++ | ++ |
| 12 | +++ | ++ | ++++ | ND | + |
| 13 | ++++ | +++ | ++ | ND | + |
| 14 | + | + | + | + | +++ |

**Table II** HNK-1 positive cells in paraffin sections of NPC

| *% of total lymphoid cells* | *Number of tumours (total = 29)* |
|---|---|
| 0–5 | 20 |
| 6–10 | 5 |
| 11–15 | 4 |

**Figure 4** Indirect immunoperoxidase staining of paraffin-embedded section of nasopharyngeal carcinoma with NHK-1 monoclonal antibody. (H&E x 1000)

HNK-1+ cells exhibited cytological features of large granular lymphocytes (LGL) (Figure 4) typical of human NK cells (Saksela *et al.*, 1979). No study on membrane T-associated antigens could be performed on these paraffin-embedded sections.

*Indirect immunofluorescence assay on cells isolated from the tumour*

Seven biopsy specimens were dissociated to get a suspension of cells. After Ficoll centrifugation, mononuclear cells were stained by immunofluorescence (Table III). On average, OKT3-positive cells were more numerous than sIg-bearing cells (44.1 ± 10.5% *vs.* 27.8 ± 6.6%). Within the T-lineage, T4+ and T8+ cells were in roughly equal proportions in three patients. T4+ cells were more numerous in two patients whereas they were virtually absent in another (patient 4). These results were in agreement with the data obtained with immunoperoxidase staining (as shown in Tables I and III). In all cases HLA-DR-positive cells were more frequent than cells carrying surface Ig (43.4 ± 12.4% *vs.* 27.8 ± 6.6%). To determine whether the HLA-DR positive sIg-negative lymphocytes expressed other lymphocyte-associated cell surface antigens, double staining assays were performed. In every test, a lymphocyte population ranging from 6 to 29% was demonstrated to coexpress the T3 and HLA-DR antigens. Furthermore a lymphocyte population ranging from 5 to 12% was demonstrated to coexpress T3 and Tac antigens in the five patients studied for this antigen. In each case tested with both OKT4 and OKT8 antibodies, the DR-positive T-cell population and the Tac-positive T-cell population included both T4 and T8 subsets in roughly equal proportion (data not shown).

No evident relationship could be found on this small population of patients when these data were analyzed with respect to the stage of the disease, lymphoproliferative responses to PHA or ConA mitogens (unpublished data), titres of antibodies to EBV-associated antigens or HLA phenotypes of the patients (Herait *et al.*, 1983; and data not shown).

### Discussion

We have undertaken a phenotypic analysis of lymphocytes present in UCNT by a combination of immunohistological staining of frozen and paraffin sections of tumour biopsies and of immunofluorescence assays on lymphocytic cells isolated from tumour specimens.

Most of the lymphoid infiltrate was found to comprise lymphocytes expressing the T3 antigen characteristic of the T-cell lineage. Among these T-lymphocytes, the distribution of T-helper/inducer and suppressor/cytotoxic cells, as defined by antibodies OKT4 and OKT8, respectively, was variable from one patient to another. Although less numerous than

**Table III** Immunofluorescence staining of mononuclear cells isolated from UCNT biopsies

| | *% positive in total mononuclear cells* | | | | | | | |
|---|---|---|---|---|---|---|---|---|
| *Patient* | *T3* | *T4* | *T8* | *HLA-DR* | *sIg* | *Tac* | *T3-HLA-DR* | *T3-Tac* |
| 4 | 65 | 2 | 55 | 45 | 13 | ND | 29 | ND |
| 5 | 50 | 30 | 20 | 20 | 10 | ND | 6 | ND |
| 6 | 29 | ND | ND | 37 | ND | 5 | 26 | 5 |
| 7 | 46 | 22 | 22 | 58 | 43 | 13 | 28 | 10 |
| 11 | 38 | 30 | 16 | 60 | ND | 10 | 19 | 7 |
| 12 | 38 | 20 | 23 | 44 | 35 | 8 | 22 | 7 |
| 13 | 43 | 24 | 28 | 40 | 38 | 11 | 27 | 12 |
| Means | 44.1 | 17.5 | 27.3 | 43.4 | 27.8 | 9.2 | 22.4 | 8.2 |

T4 positive cells in most patients, T8 positive lymphocytes tended to be predominantly located around tumour cell masses in some patients. The T4/T8 ratio was not strongly correlated with any clinical or biological feature.

Because this phenotypically-defined T8 positive subset is known to include the functional population of cytotoxic cells, it is tempting to speculate that the T8 expressing lymphocytes found in close relationship with the malignant cells might play a role in the immune reaction to the tumour. In this regard, it was striking to observe that a relatively high – although varying from one tumour to another – proportion of T3 positive cells also expressed HLA-DR molecules as detected in double staining assays. In fact 54% (range 12%–90%) of T-cells expressed HLA-DR antigens. This was in agreement with the observation of more numerous HLA-DR positive than sIg bearing cells in lymphocytes isolated from the tumour. The T10 antigen which is also found on activated T-cells (Hercend *et al.*, 1981) was also detected in all tumours tested for this marker. Whether the presence of activated T-cell associated antigens was restricted to the subpopulation expressing the T8 antigen or was rather shared by T4 positive cells as well as suggested by the preliminary results will be a matter for further study.

The reactivity of intra-UCNT lymphocytes was also studied with a monoclonal antibody directed to the receptor for interleukin-2 (IL-2) (Tac antigen) (Uchiyama *et al.*, 1981), which is only expressed on activated T-cells. Again, in all patients studied we found that a strikingly high number of T-cells (17–28%) although less numerous that T3-HLA-DR positive cells expressed the IL-2 receptor. In addition we have performed bulk cultures of these T-lymphocytes which are easily maintained in IL-2 containing medium. Clones derived from these populations will be necessary to analyze their potentially specific immune functions.

The mechanism of T-cell activation remain to be investigated. Thomas *et al.* (1984) reported the expression of HLA-DR antigens on NPC epithelial cells by immunoperoxidase labeling. We confirmed this finding in the course of this study. Furthermore we have recently found very high MHC-class II antigen expression on malignant EBNA-positive epithelial cells from nude mice-grown NPC tumours. These malignant NPC cells were shown to produce a monokine able to activate T-cell responses in the presence of mitogens, and probably similar to monocyte-derived interleukin-1 (to be reported elsewhere). NPC cells could thus share several characteristics with accessory cells able to induce T-cell activation.

EBNA is the only serologically-defined EBV-associated antigen constantly detected in malignant NPC cells. In infectious mononucleosis (IM), a benign disease caused by a polyclonal activation of B-lymphocytes stimulated by EBV, a cytotoxic immune response is based upon NK and T-cells (Lipinski *et al.*, 1979). That NK cells, as recognized by the HNK-1 monoclonal antibody, are present in UCNT tumours, might be of relevance for a potential anti-tumour activity *in situ*. In this context, EBV-specific T-cells could also play a role (Rickinson *et al.*, 1980), despite the depression of anti-EBV T-cell-mediated immunity observed elsewhere (Moss *et al.*, 1983) in the peripheral blood of NPC patients using the regression of EBV transformation assay. The target structure of EBV-specific cytotoxic T-cells is the LYDMA antigen that has not been serologically defined but whose location in the EBV genome has recently been suggested (Hennesy *et al.*, 1984). It will be interesting to find out whether UCNT cells can be killed by LYDMA-directed T-cells, and whether lymphocytes isolated from UCNT can give rise to LYDMA-specific cytotoxic T-cell clones.

In conclusion, we have reported that T-cells infiltrating UCNT demonstrate interesting phenotypic features: (i) large variation in the T4/T8 ratio, with usually an excess of T4 positive cells, but with a few striking exceptions. (ii) detection of HNK-1 positive lymphocytes, often with the morphology of LGL, sometimes in high numbers (6 to 15% of the lymphocytes in 9/29 patients), suggesting a role for NK cells in the local defence against UCNT: (iii) constant presence of T-cells expressing HLA-DR antigens, the IL-2 receptor and the T10 antigen, and therefore with the phenotype of activated T-cells.

These data suggest the existence of local immunological reactions in UCNT, involving both T- and NK cells. Further studies will be needed to elucidate their precise nature and their possible role in the control of the disease. Whether the immunological features described in this report can be related with the clinical grades and/or the prognosis of UCNT will be investigated on a large population of patients.

Supported by a Grant from the Centre National de la Recherche Scientifique (CNRS). P.H. & G.G. are recipients of IGR Research Fellowships. We thank Dr J.F. Bach, Dr E. Reinherz, Dr S. Schlossman, Dr G. Khalil, Dr M. Fellous and Dr T. Waldmann for generous gifts of monoclonal antibodies.

### References

ABO, T. & BALCH, C.M. (1981). A differentiation antigen of human NK and K cells identified by a monoclonal antibody (HNK-1). *J. Immunol.*, **127**, 1024.

CAILLAUD, J.M., BENJELLOUN, S., BOSQ, J., BRAHAM, K. & LIPINSKI, M. (1984). HNK-1-defined antigen detected in paraffin-embedded neuroectoderm tumors and those derived from the amine precursor uptake and decarboxylation system. *Cancer Res.*, **44**, 4432.

DE THÉ, G. (1980). The role of Epstein–Barr virus in human diseases: infectious mononucleosis, Burkitt's Lymphoma and nasopharyngeal carcinoma. In *Viral Oncology*, Klein, G. (ed) p. 769. Raven Press: New York.

DE THÉ, G., HO, J.H.C., ABLASHI, D.V., DAY, N.E., NACARIO, A.J.L., MARTIN-BERTHOLON, M.C., PEARSON, G. & SOHIER, R. (1975). Nasopharyngeal carcinoma. IX. Antibodies to EBNA and correlation with response to other EBV antigens in Chinese patients. *Int. J. Cancer*, **16**, 713.

DE THÉ, G., ZENG, Y., DESGRANGES, C. & PI, G.H. (1983). The existence of pre-nasopharyngeal carcinoma conditions should allow preventive interventions. In *Nasopharyngeal carcinoma: current concepts*. Prasad *et al.* (Eds) p. 365. University Malaya Press: Kuala Lumpur.

GALLILI, U., KLEIN, E., KLEIN, G., JIN, G.H. & BAL, I. (1980). Activated T lymphocytes in infiltrates and draining lymph node of nasopharyngeal carcinoma. *Int. J. Cancer*, **25**, 85.

HENLE, G. & HENLE, W. (1976). Epstein–Barr virus specific IgA serum antibodies as an outstanding feature of nasopharyngeal carcinoma. *Int. J. Cancer*, **17**, 1.

HENLE, W., HO, J.H.C., HENLE, G. & KWAN, H.C. (1973). Antibodies to Epstein–Barr virus related antigens in nasopharyngeal carcinoma: comparison of active cases and long term survivors. *J. Natl Cancer Inst.*, **51**, 361.

HENNESSY, K., FENNEWALD, S., HUMMEL, M., COLE, T. & KIEFF, E. (1984). A membrane protein encoded by Epstein–Barr virus in latent growth-transforming infection. *Proc. Natl Acad. Sci. USA*, **81**, 7207.

HERAIT, P., TURSZ, T., GUILLARD, M.Y., HANNA, K., LIPINSKI, M., MICHEAU, C., SANCHO-GARNIER, H., SCHWAAB, G., CACHIN, Y., DEGOS, L. & DE THÉ, G. (1983). HLA-A, -B and -DR antigens in North-African patients with nasopharyngeal carcinoma. *Tissue Antigens*, **22**, 335.

HERCEND, T., RITZ, J., SCHLOSSMAN, S.F. & REINHERZ, E.L. (1981). Comparative expression of T3, T10 and Ia antigens on activated human T-cell subsets. *Hum. Immunol.*, **3**, 247.

KALIL, J.E. & FELLOUS, M. (1982). Monoclonal antibodies to HLA-DR antigens. In *Ia antigens*, Vol. **2**, Ferrone, S., David, C. (eds) p. 55. CRC Press.

LIPINSKI, M., BRAHAM, K., CAILLAUD, J.M., CARLU, C. & TURSZ, T. (1983). The HNK-1 antibody detects an antigen expressed on neuroectodermal cells. *J. Exp. Med.*, **158**, 1775.

LIPINSKI, M., FRIDMAN, W.H., TURSZ, T., VINCENT, C., PIOUS, D. & FELLOUS, M. (1979). Absence of allogenic restriction in human T-cell-mediated cytotoxicity to Epstein–Barr virus infected target cells. Demonstration of an HLA-linked control at the effector level. *J. Exp. Med.*, **150**, 1310.

MOSS, D.J., CHAN, S.H., BURROWS, S.R., CHEW, T.S., KANE, R.C., STAPLES, J.A. & KUNARATNAM, N. (1983). Epstein–Barr virus specific T cell response in nasopharyngeal carcinoma patients. *Int. J. Cancer*, **32**, 301.

PEARSON, G.R., JOHANSSON, B. & KLEIN, G. (1978). Antibody-dependent cellular cytotoxicity against Epstein–Barr virus associated antigens in African patients with nasopharyngeal carcinoma. *Int. J. Cancer*, **22**, 120.

REINHERZ, E.L., KUNG, P.C., PESANDO, J.M., RITZ, J., GOLDSTEIN, G. & SCHLOSSMAN, S.F. (1979). Ia determinants on human T cell subsets defined by monoclonal antibody: activated stimuli required for expression. *J. Exp. Med.*, **150**, 1472.

RICKINSON, A. (1984). Epstein–Barr virus in epithelium. *Nature*, **310**, 99.

RICKINSON, A.B., WALLACE, M.E. & EPSTEIN M.A. (1980). HLA-restricted T-cell recognition of Epstein–Barr virus-infected B cells. *Nature*, **270**, 524.

SAKSELA, E., TIMONEN, T., RANKI, A. & HAARY, P. (1979). Morphological and functional characterization of isolated effector cells responsible for human natural killer activity to fibroblast and to cultured cell line targets. *Immunol. Rev.*, **44**, 71.

SHANMUGARATNAM, K., CHAN, S.H., DE THÉ, G., GOH, J.E.H., KHOR, T.H., SIMONS, M.J. & TYE, C.Y. (1979). Histopathology of nasopharyngeal carcinoma. Correlations with epidemiology, survival rates and other biological characteristics. *Cancer*, **44**, 1029.

THOMAS, J.A., ILIESCU, V., GRAWFORD, D.H., ELLOUZ, R., CAMMOUN, M. & DE THÉ, D. (1984). Expression of HLA-DR antigens in nasopharyngeal carcinoma: an immunohistological analysis of the tumor cells and infiltrating lymphocytes. *Int. J. Cancer*, **33**, 813.

UCHIYAMA, T., BRODER, S. & WALDMANN, T.A. (1981). A monoclonal antibody (anti-Tac) ractive with activated and functionally mature human T cells: I. Production of anti-Tac monoclonal antibody and distribution of Tac (+) cells. *J. Immunol.*, **126**: 1393.

WAKASUGI, H., BERTOGLIO, J., TURSZ, T. & FRADELIZI, D. (1985). IL-2 receptor induction on human T-lymphocytes: role for IL-2 and monocytes. *J. Immunol.*, **135**, 321.

WOLF, H. (1984). Epstein–Barr virus and carcinoma. *Nature*, **312**, 705.

WOLF, H., ZUR HAUSEN, H. & BECKER, V. (1973). EB-viral genomes in epithelial nasopharyngeal carcinoma cells. *Nature New Biol.*, **244**, 245.

ZUR HAUSEN, H., SCHULTE-HOLTHAUSEN, H., KLEIN, G., HENLE, W., HENLE, G., CLIFFORD, P. & SANTESSON, L. (1970). EBV DNA in biopsies of Burkitt tumours and anaplastic carcinomas of the nasopharynx. *Nature*, **228**, 1056.